# *Bifidobacterium longum* Ameliorates Ovariectomy-Induced Bone Loss *via* Enhancing Anti-Osteoclastogenic and Immunomodulatory Potential of Regulatory B Cells (Bregs)

**DOI:** 10.3389/fimmu.2022.875788

**Published:** 2022-05-25

**Authors:** Leena Sapra, Niti Shokeen, Konica Porwal, Chaman Saini, Asha Bhardwaj, Mary Mathew, Pradyumna K. Mishra, Naibedya Chattopadhyay, Hamid Y. Dar, Bhupendra Verma, Rupesh K. Srivastava

**Affiliations:** ^1^ Translational Immunology, Osteoimmunology & Immunoporosis Lab (TIOIL), Department of Biotechnology, All India Institute of Medical Sciences (AIIMS), New Delhi, India; ^2^ Division of Endocrinology and Centre for Research in Anabolic Skeletal Targets in Health and Illness (ASTHI), Central Drug Research Institute (CDRI), Lucknow, India; ^3^ Department of Molecular Biology, Indian Council of Medical Research-National Institute for Research in Environmental Health (ICMR-NIREH), Bhopal, India; ^4^ Division of Endocrinology, School of Medicine, Emory University Atlanta, GA, United States

**Keywords:** immunoporosis, osteoporosis, probiotics, Bregs, Tregs, Th17

## Abstract

Discoveries in the last few years have emphasized the existence of an enormous breadth of communication between osteo-immune systems. These discoveries fuel novel approaches for the treatment of several bone pathologies including osteoporosis. *Bifidobacterium longum* (BL) is a preferred probiotic of choice due to its varied immunomodulatory potential in alleviating various inflammatory diseases. Here, we evaluate the effect of BL in an ovariectomy (ovx)-induced post-menopausal osteoporotic mouse model. Our *in vitro* findings reveal that BL suppresses the differentiation and functional activity of RANKL-induced osteoclastogenesis in both mouse bone marrow cells and human PBMCs. Strikingly, BL-induced Bregs were found to be significantly more efficient in suppressing osteoclastogenesis and modulating Treg–Th17 cell balance with respect to control Bregs *in vitro*. Our *in vivo* µCT and bone mechanical strength data further confirm that BL supplementation significantly enhanced bone mass and bone strength, along with improving the bone microarchitecture in ovx mice. Remarkably, alterations in frequencies of CD19^+^CD1d^hi^CD5^+^IL-10^+^ Bregs, CD4^+^Foxp3^+^IL-10^+^ Tregs, and CD4^+^Rorγt^+^IL-17^+^ Th17 cells in distinct lymphoid organs along with serum-cytokine data (enhanced anti-osteoclastogenic cytokines IFN-γ and IL-10 and reduced osteoclastogenic-cytokines IL-6, IL-17, and TNF-α) strongly support the immunomodulatory potential of BL. Altogether, our findings establish a novel osteo-protective and immunomodulatory potential of BL in augmenting bone health under osteoporotic conditions.

## Introduction

Osteoporosis is defined as a systemic bone loss disease exemplified by deterioration of bone tissues and low bone mineral density (BMD), subsequently leading to fragility-related fractures. Osteoporosis is considered as a serious public health issue accounting for a huge socioeconomic burden. Postmenopausal osteoporosis mainly stems from the cessation of ovarian function and decline in estrogen that stimulates bone resorption and rapid bone loss (1). Although currently available therapies to treat osteoporosis are effective, side effects (real and perceived) are an impediment to treatment adherence by the patients. Nevertheless, various dietary supplements hold promise in conserving bone mass in postmenopausal condition.

“Immunoporosis” is an emerging area of research that studies the involvement of the immune system in osteoporosis (2). In this regard, the roles of regulatory T cells (Tregs) and inflammatory T cells (Th17) in bone homeostasis have been amply elucidated. Recently, our group along with others has reported that the imbalance between Treg and Th17 cells is key to the pathophysiology of bone-related diseases including osteoporosis (2, 3). Th17 cells are an established osteoclastogenic subset of T cells owing to the production of osteoclastogenic cytokines including IL-6, TNF-α, IL-17, and RANKL (4, 5). On the contrary, Tregs are anti-osteoclastogenic T-cell subsets that produce anti-osteoclastogenic cytokines including IL-10 and TGF-β. CD4^+^CD25^+^Foxp3^+^ Tregs suppress osteoclastogenesis and bone-resorptive functions of osteoclasts *via* TGF-β1 and IL-10 in human peripheral blood mononuclear cells (PBMCs) (6). Recently, we demonstrated that regulatory B cells (Bregs) suppressed osteoclastogenesis *in vitro* and protected ovariectomy (ovx) mice from bone loss, thus underscoring the importance of this novel subset of cells in mitigating osteoporosis (7). Bregs are further reported to modulate the differentiation of Tregs and Th17 cells in autoimmune diseases including type 1 diabetes (T1D), systemic lupus erythematosus (SLE), and rheumatoid arthritis (RA) (8). A recent study showed that the adoptive transfer of Bregs into osteoporotic mice successfully reduced the deterioration in alveolar bone by lowering the percentage of IL-17-producing Th17 cells (9). Thus, pharmacological modulation of Bregs, Tregs, and Th17 cells could represent a novel bone anabolic therapy for osteoporosis.

In recent years, the “gut–immune–bone” axis has gained much attention from researchers worldwide. In humans, approximately 100 trillion microbes reside with the major fraction residing in the gastrointestinal tract. According to the WHO guidelines, probiotics are viable microorganisms that impart beneficial health effects when administered in adequate amounts. Various studies have demonstrated that manipulation of the gut microbiota *via* administration of probiotics enhances bone heath (10–12). One of the widely studied multifunctional probiotic strains of *Bifidobacterium* species is *Bifidobacterium longum* (BL). It is a gram-positive, anaerobic bacterium and among the first microbes to colonize the human gastrointestinal (GI) tract and modulate the entire gut microbial diversity. BL is found to be effective in alleviating gastrointestinal and infectious diseases by stabilizing the gut microbiota and intestinal environment (13). A study reported that BL administration enhanced the BMD of ovx rats by upregulating the expression of the bone morphogenetic protein-2 (BMP-2) gene (14). *Bifidobacterium* also alters the gut microbiota, thereby modulating the metabolism of Tregs (15). However, there is no study that delineates the immunomodulatory role of BL and its impact on augmenting bone health in osteoporosis till date. Thus, in the present study, we investigated the immunoporotic potential of BL in enhancing bone health in a postmenopausal osteoporotic (ovx) mouse model.

To our knowledge, this is the first study that reveals the osteo-protective and immunomodulatory potential of BL in an ovx mouse model *via* its ability to significantly enhance both the anti-osteoclastogenic and immunomodulatory potential of BL-induced Bregs. Our both *in vitro* and *in vivo* data strongly suggest that BL-induced Bregs exhibit significantly enhanced potential of suppressing osteoclastogenesis along with modulating Treg and Th17 differentiation *in vitro*. Of note, the immunomodulatory potential of BL is further strengthened from our serum cytokine data where we observe enhanced levels of anti-osteoclastogenic cytokines, i.e., IL-10 (signature cytokine of Breg and Tregs) and IFN-γ together with reduced levels of osteoclastogenic cytokines, i.e., TNF-α, IL-6, and IL-17 (signature cytokine of Th17 cells). Altogether, the present study underlines the osteoprotective role of BL *via* modulating the immunoporotic “Breg–Treg–Th17 cell axis,” thereby opening novel avenues for both the treatment and management of inflammatory bone loss observed in postmenopausal osteoporosis.

## Material and Methods

### Reagents and Antibodies

The following antibodies/kits were procured from eBioscience (San Diego, CA, USA): PerCp-Cy5.5 Anti-Mouse-CD4-(RM4-5) (550954), APC Anti-Mouse/Rat-Foxp3 (FJK-16s) (17-5773), PE Anti-Human/Mouse-Rorγt (AFKJS-9) (12-6988), PerCp-Cy5.5 Anti-Mouse-CD19 (1D3) (45-0193-82), PE-Cy7 Anti-Mouse-CD5 (53-7.3) (25-0051-81), APC Anti-Mouse-CD1d (1B1) (17-0011-82), APC Cy7 Anti-Mouse-F4/80-(BM8) (47-4801-82), Foxp3/Transcription factor staining buffer (0-5523-00), and RBC lysis buffer (00-4300-54). The following ELISA kits were brought from R&D: Mouse IL-10 (M1000B) and Mouse IL-17 (M1700) Quantikine ELISA kits. The following ELISA kits and reagents were brought from BD (Franklin Lakes, NJ, USA): Mouse IL-6 (OptEIA™-555240) and Mouse TNF-α (OptEIA™-560478). Acid phosphatase, leukocyte (TRAP) kit (387A), FITC-phalloidin (P5282), and DAPI were purchased from Sigma (St. Louis, MO, USA). Macrophage-colony stimulating factor (M-CSF) (300-25) and receptor activator of nuclear factor κB-ligand (sRANKL) (310-01), Human TGF-β1 (AF-100-21C), Murine IL-2 (AF-212-12), Murine IL-6 (AF-216-16), and Murine IL-23 (200-23) were procured from PeproTech (Rocky Hill, NJ, USA). α-Minimal essential media and RPMI-1640 were purchased from Gibco (Grand Island, NY, USA). Bifido broth was procured from HiMedia Labs (Hyderabad, India). *Bifidobacterium longum* UBBL-64 was procured from Unique Biotech Ltd., Hyderabad, India.

### Animals

All *in vitro* and *in vivo* experiments were carried out in 8–10-week-old female C57BL/6 J mice. Mice were maintained under specific pathogen-free (SPF) conditions at the animal facility of All India Institute of Medical Sciences (AIIMS), New Delhi, India. Mice were fed with sterilized food and autoclaved drinking water *ad libitum*. Mice were exposed to bilateral ovariectomy (ovx), and sham surgery was performed on mice after anesthetizing mice with ketamine (100–150 mg/kg) and xylazine (5–16 mg/kg) intraperitoneally. Subsequently, mice were randomly allocated into three groups with 6 mice in each group, i.e., sham (control), ovx, and ovx + *Bifidobacterium longum* (BL). After 1 week post-surgery, the ovx+BL group of mice were orally gavaged with 400 µl of BL suspension (containing 10^9^ cfu) daily in drinking water for a period of 6 weeks. At the end of 6 weeks, animals were euthanized, and blood, bones, and lymphoid tissues were harvested for further analysis. The body weight of mice was documented at regular intervals (day 1, day 21, and day 45) during the dose administration period. All the measures were performed after the due approval of the protocols submitted to the Institutional Animal Ethics Committee of AIIMS, New Delhi, India (196/IAEC-1/2019).

### *Bifidobacterium longum* Bacterial Culture

*Bifidobacterium longum* UBBL-64 (M1395) was cultured in Bifido broth containing 0.05% of L-cysteine under anaerobic conditions. On the following day, overnight culture was subcultured into fresh Bifido broth containing freshly prepared L-cysteine (0.05%), and the culture was grown until it attained the log phase (OD 600nm = 0.4). Next, cells were harvested and washed with 1× PBS twice to remove the traces of Bifido broth with centrifugation at 4,000 rpm for 10 min and proceeded for conditioned medium preparation. The conditioned media (CM) of *B. longum* were prepared by resuspending the cells with either α-MEM or RPMI-1640 antibiotic-free media and incubated for the next 3 h at 37°C. Lastly, cell-free BL-CM were collected *via* pelleting out the bacterial cells, pH neutralized, and filtered with a 0.22-µm filter. Further, BL-CM were used for all downstream assays, e.g., osteoclast and immune cell differentiation.

### Osteoclast Differentiation and TRAP Staining

For osteoclast differentiation, mouse bone marrow cells (BMCs) were isolated from 8- to 12-week-old C57BL/6J mice by flushing the femoral bone with complete α-MEM media. After performing RBC lysis with 1× RBC lysis buffer, BMCs were cultured overnight in a T-25 flask in endotoxin-free complete α-MEM media (10% heat-inactivated FBS) supplemented with M-CSF at the 35-ng/ml concentration. The next day, non-adherent cells were collected and seeded in 96-well plates (50,000 cells/well) in complete α-MEM media supplemented with M-CSF (30 ng/ml) and RANKL (100 ng/ml) in the presence or absence of BL-CM at different ratios, viz., 1:100, 1:10, and 1:5 for 4 days. On day 3, half media were replenished with fresh complete α-MEM media supplemented with fresh factors. Lastly, for monitoring the generation of multinucleated osteoclasts, tartrate-resistant acid phosphatase (TRAP) staining was carried out according to the manufacturer’s instructions. Briefly, at the end of incubation, cells were carefully washed thrice with 1× PBS and fixed with a fixative solution containing citrate, acetone, and 3.7% formaldehyde solution and incubated for 10 min at RT. Next, fixed cells were washed twice with 1× PBS and incubated with a TRAP-staining solution in the dark at 37°C for 5–15 min. Multinucleated TRAP-positive cells with ≥3 nuclei were considered as osteoclasts, and these cells were counted and imaged using an inverted microscope (Eclipse, TS100, Nikon and EVOS, Thermo Scientific, Waltham, MA, USA). The area of osteoclasts was estimated with ImageJ software (NIH, USA).

### F-Actin Ring Formation Assay

F-actin ring formation assay was carried out as described previously (16). Briefly, bone marrow-derived osteoclast precursors were seeded on glass coverslips in 12-well plates; at day 4, processing for F-actin polymerization staining was performed. Cells were washed twice with 1× PBS and fixed with 4% paraformaldehyde (PFA) for 20 min and permeabilized with 0.1% Triton X-100 for 5 min. Further, to block the non-specific binding, cells were blocked with 1% BSA for 30 min and stained with FITC-labeled phalloidin for 1 h at RT in the dark. Lastly, cells were stained with DAPI (10 µg/ml) for 5 min in the dark. Finally, slides were observed under an immunofluorescence microscope (Imager.Z2, Zeiss, Jena, Germany) for analyzing F-actin ring formation.

### B-Cell Purification and Activation

Splenic B cells from C57BL/6 mice were purified by magnetic separation described previously (7). Briefly, following RBC lysis, cells were subjected to a biotin-labeled CD19 antibody (BD, USA) and incubated for 30 min at 4°C. After washing, the labeled cells were incubated with Streptavidin Particles Plus-DM for 30 min at 4°C. Further, cells underwent magnetic separation and positive/negative fractions having B-cell purity (>95%) were cultured in 24-well plates (2 × 10^6^) in the presence or absence of LPS (10 µg/ml) and BL-CM (1:5) for 24 h at 37°C in a 5% CO_2_ incubator. At the end of incubation, LPS and LPS + BL-CM-induced Bregs were harvested and processed for either flow cytometry or coculturing experiments (with BMCs for osteoclastogenesis and naïve T cells for Treg/Th17 cell differentiation).

### Coculture of Bregs With BMCs and Naïve T Cells

For estimating the potential of BL-CM-induced Bregs to suppress osteoclast differentiation, BMCs were cocultured with either Bregs or BL-CM-induced Bregs in 96-well plates in different ratios (10:1, 5:1, and 1:1) in the presence of M-CSF (30 ng/ml) and RANKL (100 ng/ml) for 4 days. At an interval of 2 days, half media were replenished with media containing fresh factors. After 4 days of incubation, TRAP staining was performed to evaluate the generation of osteoclasts. For evaluating the potential of Bregs to modulate Treg–Th17 differentiation, BL-CM-induced Bregs were cocultured with negatively selected naïve T cells (CD4^+^CD25^-^ T cells) at 1:1 in anti-CD3 (10 µg/ml) and anti-CD28 (2 µg/ml) mAb-coated 48-well plates under non-polarization conditions. On day 4, cells were harvested, and flow cytometry was performed for estimating the percentages of CD4^+^Foxp3^+^IL-10^+^ Tregs and CD4^+^Rorγt^+^IL-17^+^ Th17 cells.

### Osteoclast Differentiation From Human PBMCs

PBMCs were obtained from heparinized blood by gently layering the blood on Histopaque at 1:3 in a 15-ml tube and centrifuged at 800 × g for 25 min at RT (with brakes off). The buffy coat layer beneath the plasma was carefully collected in a separate tube and washed with 1× PBS by inverting the tube gently and centrifuging the tube at 400 × g for 10 min at 4°C. The obtained PBMCs were seeded in a 96-well plate at a seeding density of 1× 10^6^ cells/well, and the plate was incubated for 2 h in a humidified 5% CO_2_ incubator and washed with α-MEM twice (without FBS). Upon adherence, cells were incubated with α-MEM supplemented with 10% FBS, M-CSF (30 ng/ml), and RANKL (100 ng/ml) in the presence or absence of BL-CM at different ratios (1:100, 1:10, and 1:5), and the plate was incubated for the next 14 days in a CO_2_ incubator with half-medium replenishment on every 3rd day (i.e., 72 h). At the end of incubation, TRAP staining was performed for evaluating the differentiation of multinucleated osteoclasts. All the measures were performed after the due approval of the protocols submitted to the Institute Ethics Committee for Post Graduate Research (IECPG-482), AIIMS, New Delhi, India.

### Scanning Electron Microscopy

Scanning electron microscopy (SEM) was performed for the cortical region of femoral bones, as described previously (10, 16, 17). Briefly, bone samples were kept in 1% Triton-X-100 for 2–3 days and later samples were transferred to 1× PBS buffer till the final analysis was performed. Next, after the preparation of bone slices, the samples were dried under the incandescent lamp and sputter coating was done. Afterward, bones were scanned in a Leo 435 VP microscope equipped with a 35-mm photography system. SEM images were digitally photographed at ×100 magnification to catch the finest cortical region. After imaging, SEM images were further analyzed by MATLAB (MathWorks, Natick, MA, USA).

### Atomic Force Microscopy

Upon drying femur bones under 100-W lamps for 6 h followed by high-vacuum drying, samples were analyzed using an atomic force microscope (AFM) (Innova Icon, Bruker, Billerica, MA, USA) set in Acoustic AC mode. This was assisted by cantilever (NSC 12(c) MikroMasch, Silicon Nitride Tip) and NanoDrive version 8 software, set at a constant force of 0.6 N/m with a resonant frequency at 94–136 kHz. Images were recorded at a scan speed of 1.5–2.2 lines/s in the air at room temperature. Images were later processed and analyzed by using Nanoscope analysis software.

### Micro-Computed Tomography Measurements

Micro-computed tomography (µ-CT) scanning and analysis were performed using *in vivo* X-ray SkyScan 1076 scanner (Aartselaar, Belgium) tomography, as described before (18). Briefly, scanning was done at 50 kV, 204 mA, using a 0.5-mm aluminum filter by positioning the samples at the right orientation in the sample holder. For the reconstruction process, NRecon software was employed. After reconstruction, ROI was drawn at a total of 100 slices in secondary spongiosa at 1.5 mm from the distal border of growth plates and further processed for CTAn software for evaluating and calculating the micro-architectural parameters of bone samples. Several 3D-histomorphometric parameters were obtained, viz., bone volume/tissue volume (BV/TV), trabecular thickness (Tb.Th), trabecular separation (Tb.Sp), etc. The volume of interest of u-CT scans made for trabecular and cortical regions was used to determine the BMD of LV5, femur, and tibia. BMD was measured by using hydroxyapatite phantom rods of 4-mm diameter with known BMD (0.25 g/cm^3^ and 0.75 g/cm^3^) as a calibrator (16).

### Bone Strength Testing

To measure the biomechanical properties of bones, femoral bones of mice in all the respective groups were exposed to three-point bending by employing the bone strength tester model TK-252C/RDT (Muromachi Kikai Co. Ltd., Tokyo, Japan). Briefly, for this, femoral bone was placed on the two supports that were kept at a constant distance of 1 cm. By means of load displacement curves, the following bone mechanical strength parameters were evaluated: maximum power (N), energy to fracture (mJ), and stiffness (N/mm).

### Flow Cytometry

Cells were harvested from various lymphoid organs (viz., BM and spleen) and stained with antibodies specific for macrophages, Bregs, Tregs, and Th17 cells. For the macrophage panel, BM cells were stained with the anti-F4/80-APC Cy7 antibody. Breg/Treg/Th17 cells were first stained for Bregs (anti-CD19-PerCP-Cy5.5, anti-CD5-PE-Cy7, and anti-CD1d-APC antibodies) and Tregs/Th17 (anti-CD4-PerCP-Cy5.5) antibodies for cell surface staining and incubated for 30 min in the dark on ice. After washing, cells were further fixed and permeabilized with a 1× fixation–permeabilization buffer for 30 min on ice in the dark. Finally, for intracellular staining, cells were stained with anti-IL-10-BV421 (Bregs), anti-Foxp3-APC & anti-IL-10-BV421 (Tregs) and anti-Rorgt-PE & anti-IL-17-APC (Th17 cells) for 45 min. After washing, cells were acquired on BD LSRFortessa (USA). FlowJo 10 (Tree Star, Woodburn, OR, USA) software was used to analyze the samples, and the gating strategy was done as per experimental requirements.

### Enzyme-Linked Immunosorbent Assay

Enzyme-linked immunosorbent assay (ELISA) was carried out for the quantitative assessment of cytokines IL-6, IL-10, IL-17, TNF-α, and IFN-γ in blood sera of all the mouse groups by utilizing commercially available kits as per the manufacturer’s instructions.

### Statistical Analysis

Statistical differences between the distinct groups were evaluated by employing analysis of variance (ANOVA) with subsequent analysis *via* Student’s t-test paired or unpaired as appropriate. All the values in the data are expressed as mean ± SEM (n = 6). Statistical significance was determined as p ≤ 0.05 (*p < 0.05, **p < 0.01, ***p, 0.001) with respect to the indicated groups.

## Results

### BL Suppress Differentiation and Function of Osteoclasts

**Figure 1:**
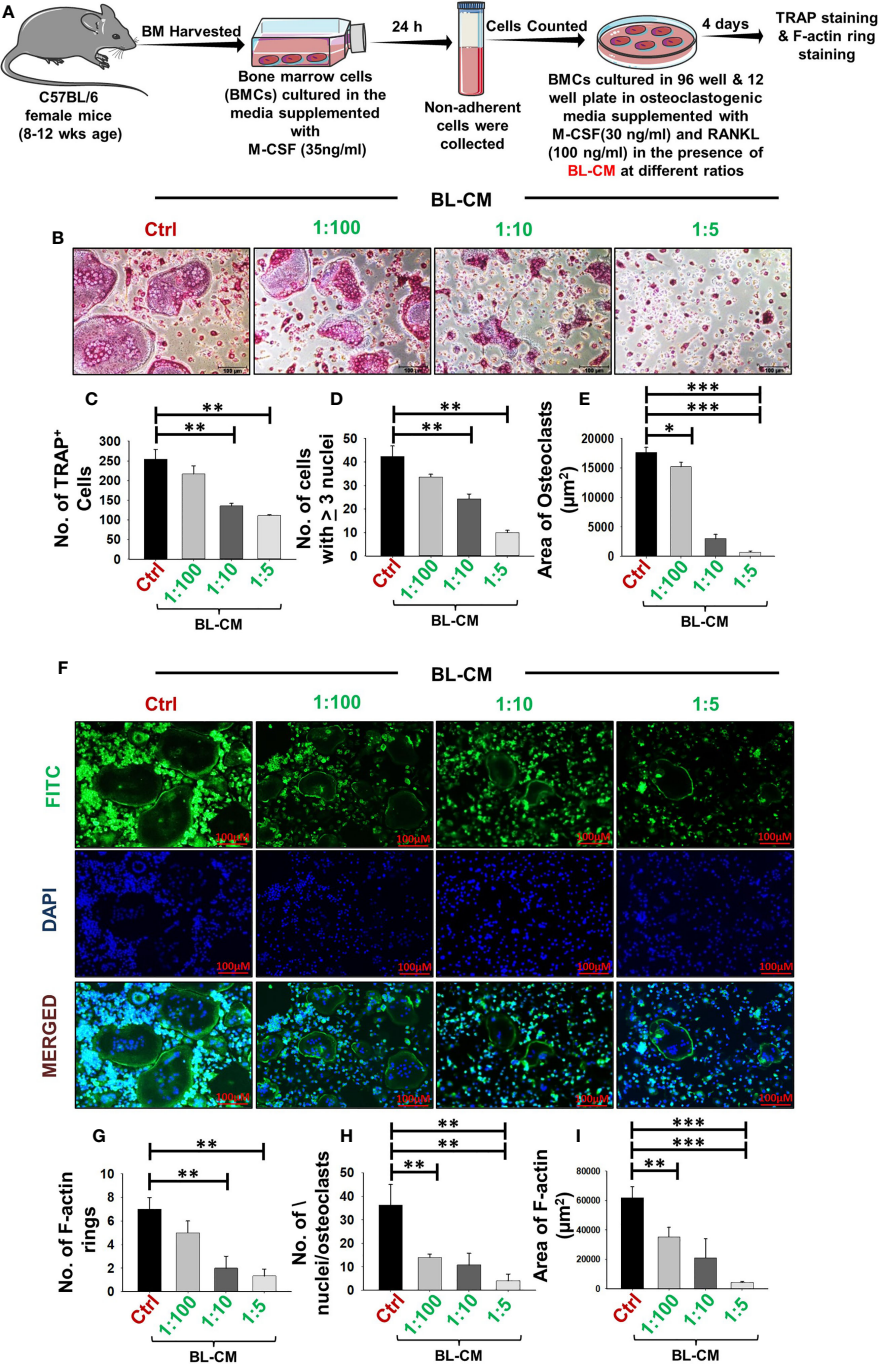
BL suppress osteoclastogenesis and F-actin polymerization in a dose-dependent manner: **(A)** Osteoclast differentiation was induced in bone marrow cells (BMCs) with M-CSF (30 ng/ml) and RANKL (100 ng/ml) with or without *Bifidobacterium longum*-conditioned media (BL-CM) at different ratios of 1:100, 1:10, and 1:5 for 4 days. Giant multinucleated cells were stained with TRAP, and cells with ≥ 3 nuclei were considered as mature osteoclasts. **(B)** Photomicrographs at ×20 magnification were taken. **(C)** Number of TRAP-positive cells. **(D)** Number of TRAP-positive cells with more than 3 nuclei. **(E)** Area of osteoclasts. **(F)** F-Actin and nuclei were stained with FITC-conjugated phalloidin and DAPI, respectively. Images were captured in a fluorescence microscope (Imager.Z2 Zeiss microscope) at ×10 magnification. **(G)** Number of F-actin rings. **(H)** Number of nuclei per osteoclasts. **(I)** Area of the F-actin ring. The above images are indicative of one independent experiment, and similar results were obtained in at least three independent experiments (n ≥ 3). Statistical significance was considered as p  ≤  0.05 (*p  ≤  0.05, **p  ≤  0.01, ***p  ≤  0.001) with respect to indicated groups.

To elucidate the involvement of BL in modulating bone health, we initially examined the potential of BL on receptor activator of nuclear kappa B ligand (RANKL)-induced osteoclast differentiation and their functional activity. A schematic diagram presents the experimental protocol (**Figure 1A**; for details, refer to *Methods*). BMCs were stimulated with osteoclastogenic media supplemented with M-CSF (30 ng/ml) and RANKL (100 ng/ml) in the presence or absence of BL-CM at different ratios (1:100, 1:10, and 1:5). After 4 days, cells were harvested and processed for both TRAP staining and F-actin ring polymerization assay. There was a significant reduction in the differentiation of osteoclasts in a dose-dependent manner as evident by the number of multinucleated (>3 nuclei) TRAP-positive cells in the BL-treated group in comparison to the control group (**Figures 1B–D**). Furthermore, the area of multinucleated osteoclasts in treatment groups was significantly less than in the control group (**Figure 1E**). Also, the number and area of F-actin rings were significantly decreased, which indicated that BL inhibited not only osteoclastogenesis but also the functional activity of osteoclasts (**Figures 1F–I**).

### BL Modulates Differentiation of Bregs

**Figure 2:**
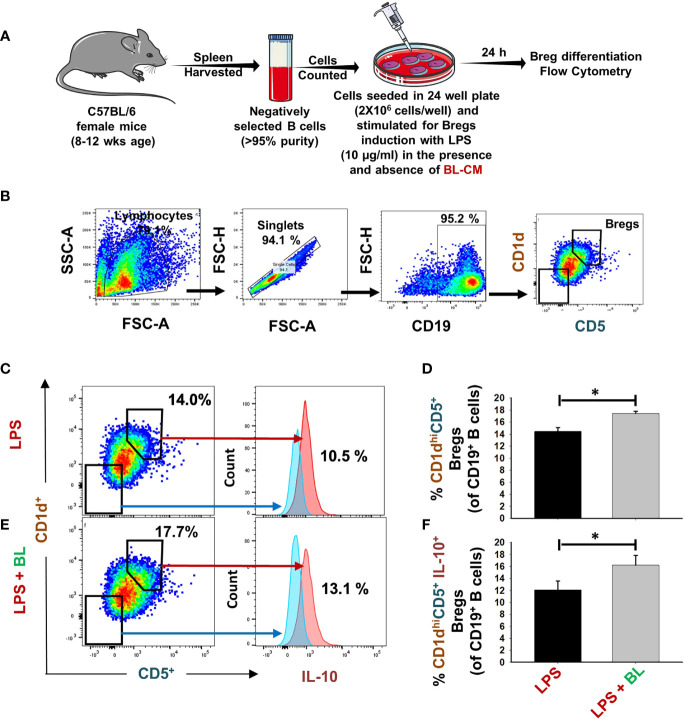
BL enhances differentiation of IL-10-producing Bregs: **(A)** For Bregs stimulation, splenic CD19^+^ B cells were positively/negatively selected and stimulated with LPS (10 µg/ml) in the presence and absence of BL-CM at 1:5 dilution. After 24 h, cells were analyzed for CD1d CD5 and IL-10 expression by FACS. **(B)** Gating strategy followed for data analysis. **(C)** Dot plots and histograms depicting the percentages of CD19^+^CD1d^high^CD5^+^ Bregs and CD19^+^CD1d^high^CD5^+^IL-10^+^Bregs in control. **(D)** Bar graphs depicting the percentages of CD19^+^CD1d^high^CD5^+^ Bregs. **(E)** Dot plots and histograms depicting the percentages of CD19^+^CD1d^high^CD5^+^ Bregs and CD19^+^CD1d^high^CD5^+^IL-10^+^Bregs in BL. Bregs. **(F)** Bar graphs depicting the percentages of CD19^+^CD1d^high^CD5^+^IL-10^+^Bregs. The above images are indicative of one independent experiment, and similar results were obtained in at least three independent experiments (n ≥ 3). Statistical significance was considered as p  ≤  0.05 (*p  ≤  0.05) with respect to indicated groups.

Recently, our group reported that Bregs exhibit the potential to inhibit osteoclast differentiation (7). Thus, we were interested in estimating the potential of BL in modulating the differentiation of Bregs. For this purpose, positively selected splenic B cells were stimulated under Breg-polarizing conditions in the presence of BL-CM for 24 h (**Figure 2A**). We observed that BL significantly enhanced the percentage of CD19^+^CD1d^hi^CD5^+^ Bregs (p < 0.05) along with a significant increase in IL-10 production (**Figures 2B–F**). Taken together, these results indicate a strong immunomodulatory potential of BL in modulating Breg differentiation.

### BL-Stimulated Bregs Have Enhanced Anti-Osteoclastogenic Potential

**Figure 3:**
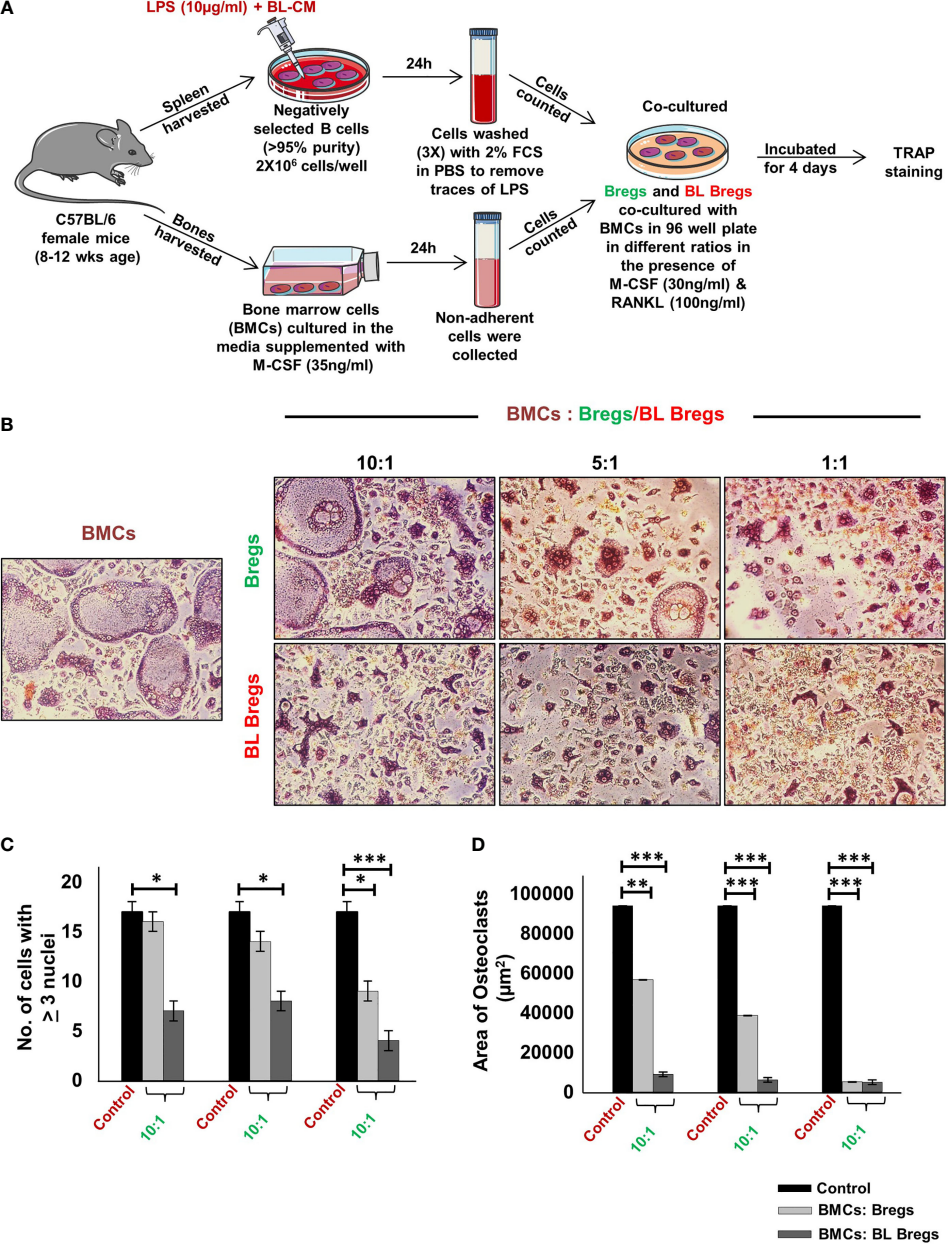
BL enhances anti-osteoclastogenic potential of Bregs: **(A)** BMCs and LPS stimulated and LPS + BL-CM were cocultured in a cell culture plate in the presence of M-CSF (30 ng/ml) and RANKL (100 ng/ml) for 4 days. B cells were induced with LPS (10 µg/ml) and BL-CM (1:5) for 24 h prior to cocultures. **(B)** LPS + BL-CM-induced Bregs suppress osteoclastogenesis more efficiently in comparison to LPS-induced Bregs. **(C)** Number of TRAP-positive cells with more than 3 nuclei. **(D)** Area of osteoclasts. The above images are indicative of one independent experiment, and similar results were obtained in at least three independent experiments (n ≥ 3). Statistical significance was considered as p  ≤  0.05 (*p  ≤  0.05, **p  ≤  0.01, ***p ≤  0.001) with respect to indicated groups.

Next, we studied whether BL has the potential to further enhance the anti-osteoclastogenic potential of Bregs. To this aim, we cocultured BL-induced Bregs and BMCs at various ratios (10:1, 5:1, and 1:1) for 4 days (**Figure 3A**). Our TRAP data showed that BL-induced Bregs inhibited the generation of osteoclasts more efficiently than the non-BL-stimulated Bregs (**Figures 3B–D**). These data indicate that BL could also prevent bone loss by suppressing osteoclast differentiation *via* Bregs.

### BL-Stimulated Bregs Are Robust Regulators of Treg–Th17 Cell Differentiation

**Figure 4:**
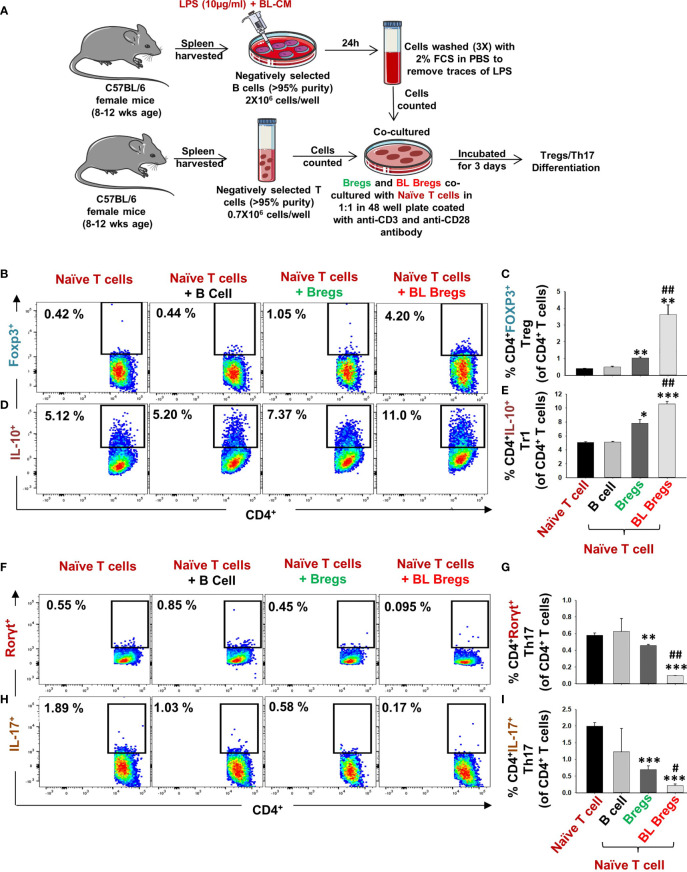
BL enhances the immunomodulatory potential of Bregs: **(A)** Naïve T cells and LPS-stimulated and LPS + BL-CM were cocultured in anti-CD3 and CD28 coated plate for 3 days. B cells were induced with LPS (10 µg/ml) and BL-CM (1:5) for 24 h prior to cocultures. **(B)** Dot plots depicting the percentages of CD4^+^FOXP3^+^ Tregs. **(C)** Bar graphs representing the percentages of CD4^+^FOXP3^+^ Tregs. **(D)** Dot plots depicting the percentages of CD4^+^IL-10^+^ Tr1 cells. **(E)** Bar graphs representing the percentages of CD4^+^IL-10^+^ Tr1 cells. **(F)** Dot plots depicting the percentages of CD4^+^Rorγt^+^Th17 cells. **(G)** Bar graphs representing the percentages of CD4^+^Rorγt^+^Th17 cells. **(H)** Dot plots depicting the percentages of CD4^+^IL-17^+^ Th17 cells. **(I)** Bar graphs representing the percentages of CD4^+^IL-17^+^ Th17 cells. (* denotes comparison of the indicated group with respect to naïve T cells, and # denotes comparison of the indicated group with respect to the Bregs group). The above images are indicative of one independent experiment, and similar results were obtained in at least three independent experiments (n ≥ 3). Statistical significance was considered as p  ≤  0.05 (*p  ≤  0.05, **p  ≤  0.01, ***p  ≤  0.001) with respect to indicated groups (*, ** and *** indicated comparison between Naive T cells and Bregs ; # and ## indicate comparison between Bregs and BL-Bregs).

Moving ahead in our study, we were next keen in assessing the immunomodulatory potential of BL-induced Bregs in regulating the differentiation of naïve T cells into either Tregs or Th17 cells. To this aim, we cocultured BL-stimulated Bregs with negatively selected naïve T cells at a 1:1 ratio for 3 days (**Figure 4A**) in anti-CD3- and anti-CD28-coated plates under non-polarizing conditions. Subsequently, cells were harvested and analyzed for the percentages of CD4^+^Foxp3^+^IL-10^+^ Tregs and CD4^+^Rorγt^+^IL-17^+^ Th17 cells by flow cytometry. We observed that BL-stimulated Bregs significantly increased the percentage of both CD4^+^Foxp3^+^ Tregs (p < 0.01) and CD4^+^IL-10^+^ Tr1 cells (p < 0.001) in comparison to control groups (**Figures 4B–E**). Besides, BL-stimulated Bregs significantly reduced the percentage of CD4^+^Rorγt^+^IL-17^+^ Th17 cells (p < 0.001) (**Figures 4F–I**). Taken together, these data strongly suggest that the enhancement in the percentage of Bregs in response to BL stimulation is pivotal for the efficient induction of anti-osteoclastogenic Tregs along with concurrent inhibition of osteoclastogenic Th17 cells, thereby pointing toward the role of the “Breg–Treg–Th17” cell axis in ameliorating bone loss *in vivo*.

### BL Ameliorates Bone Loss Under Postmenopausal Osteoporotic Conditions

**Figure 5:**
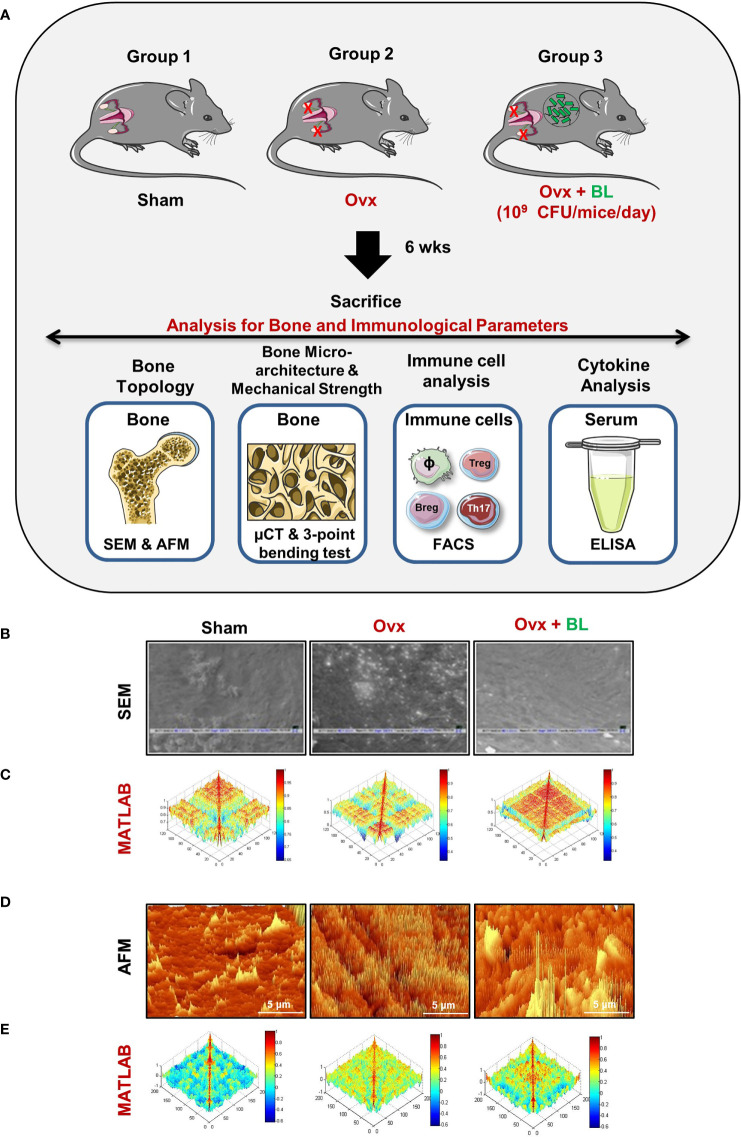
BL administration attenuates bone loss in Ovx mice: **(A)** Experimental layout followed for *in vivo* studies. Mice were divided into 3 groups, viz., sham, Ovx, and Ovx + BL groups, that received BL at 10^9^ CFU/day orally reconstituted in drinking water. At the end of 45 days, mice were sacrificed and analyzed for various parameters. **(B)** 2D SEM images. **(C)** 2D MATLAB analysis of SEM images. **(D)** 3D AFM images. **(E)** 3D MATLAB analysis of AFM images. The representative images are indicative of one independent experiment, and comparable results were obtained in two different independent experiments with n =6 mice/group/experiment.

We next assessed the effect of BL in mitigating bone loss caused by estrogen deficiency (ovx) conditions using adult female C57BL/6 mice that were distributed into three groups: sham surgery (ovary intact), bilateral ovx (both ovaries removed), and ovx group with a daily oral administration of BL (10^9^cfu) for 6 weeks. At the end of the treatment, mice were sacrificed by euthanasia, and the bones were collected for further studies (**Figure 5A**). There was no significant difference in the bodyweight between the groups throughout the duration of the study (**Figure S1**). SEM of the femoral cortical bone sections showed higher bone resorption pits or lacunae in the ovx group compared with the sham group whereas the BL group showed a significant mitigation of pits compared with the ovx group (**Figure 5B**). To further examine these 2D SEM images quantitatively in a more statistical manner, we performed MATLAB analysis to obtain the association between bone loss and bone mass. MATLAB analysis of SEM images represents the magnitude of homogeneity where the higher correlation is denoted by the red color (enhanced bone mass) and the blue color (lower correlation) symbolizes enhanced bone loss. MATLAB analysis of 2D SEM images indicated that the BL-administered ovx group showed a higher correlation value and thus enhanced bone mass (**Figure 5C**). We next estimated the 3D topology of the bone surface in response to BL treatment by AFM which indicated reduced bone resorption and roughness of the bone surface in the BL-administered ovx group (**Figure 5D**). Moreover, MATLAB analysis of AFM images further suggests improved bone architecture and decreased osteoclastogenesis in the BL-treated group compared with the ovx group (**Figure 5E**). These findings strongly indicate that BL administration attenuates bone loss in ovx mice.

### BL Maintains Bone Microarchitecture in ovx Mice

**Figure 6:**
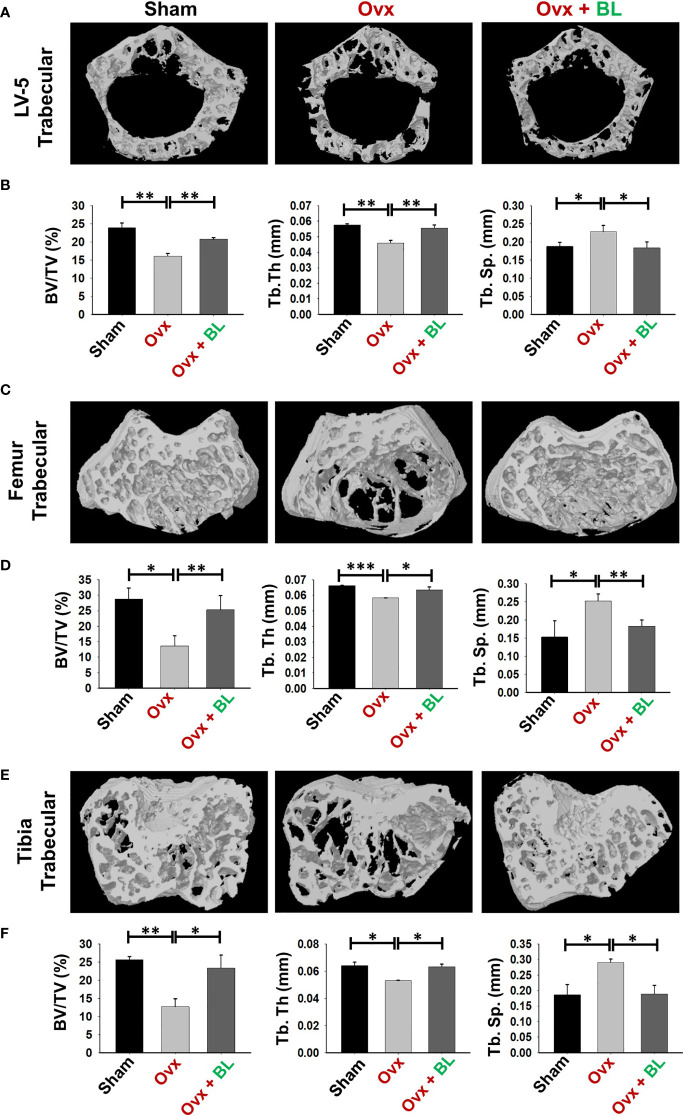
BL administration improves trabecular bone microarchitecture. 3D uCT reconstruction of LV-5 trabecular, femur trabecular, and tibia trabecular of all groups. **(A)** Bone micro-architecture of LV-5 trabecular. **(B)** Histomorphometric parameters of LV-5 trabecular. **(C)** Bone micro-architecture of femur trabecular. **(D)** Histomorphometric parameters of femur trabecular. **(E)** Bone micro-architecture of tibia trabecular. **(F)** Histomorphometric parameters of tibia trabecular. Histomorphometric parameters: BV/TV, bone volume/tissue volume ratio; Tb. Th., trabecular thickness; Tb. Sp., trabecular separation. The results were evaluated by ANOVA with subsequent comparisons by Student’s t-test for paired or non-paired data. Values are reported as mean ± SEM. The above graphical representations are indicative of one independent experiment, and similar results were obtained in two different independent experiments with n = 6. Statistical significance was considered as p ≤ 0.05 with respect to indicated mouse groups.

Osteoporosis and fracture risk are high in both spine and hip of postmenopausal women. These two anatomical regions are represented by lumbar vertebra (5th) and proximal femur metaphysis respectively. Micro-computed tomography (µ-CT) assessment of lumbar vertebrae (LV)-5 showed loss of bone volume and deteriorated microarchitecture in the ovx mice compared with both sham and BL-treated groups (**Figure 6A**). BL supplementation significantly enhanced bone volume per tissue volume (BV/TV) (p < 0.01) and trabecular thickness (Tb.Th) (p < 0.01) and decreased trabecular separation (Tb.Sp) (p < 0.05) compared with the ovx group (**Figure 6B**).

**Figure 7:**
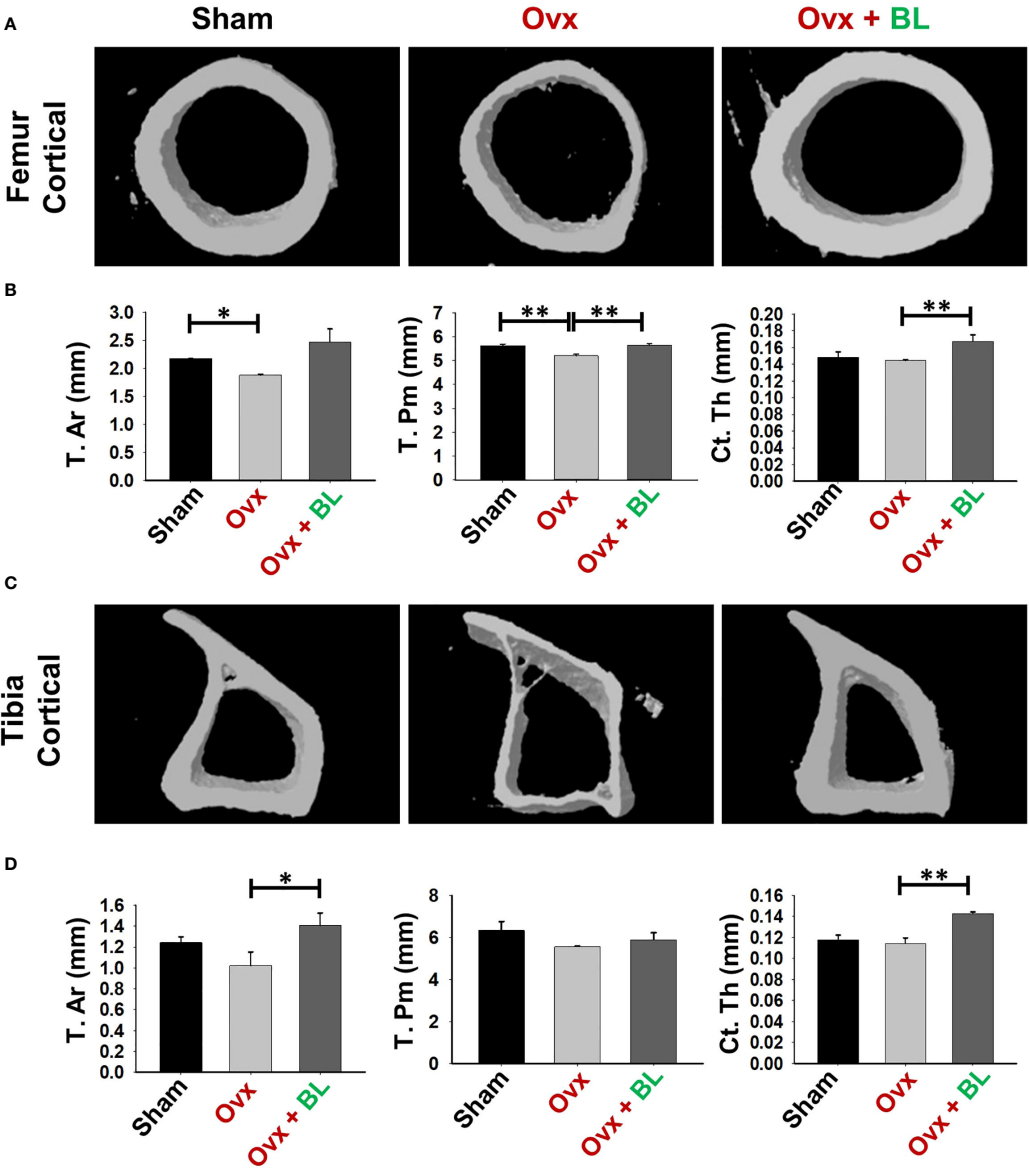
BL administration improves cortical bone microarchitecture. 3D uCT reconstruction of femur cortical and tibia cortical of all groups. **(A)** Bone micro-architecture of femur cortical. **(B)** Histomorphometric parameters of femur cortical. **(C)** Bone micro-architecture of tibia cortical. **(D)** Histomorphometric parameters of tibia cortical. Histomorphometric parameters of tibia cortical. Tt. Ar., total cross-sectional area; T. Pm., total cross-sectional perimeter; Ct. Th., cortical thickness. The results were evaluated by ANOVA with subsequent comparisons by Student’s t-test for paired or non-paired data. Values are reported as mean ± SEM. The above graphical representations are indicative of one independent experiment, and similar results were obtained in two different independent experiments with n = 6. Statistical significance was considered as p ≤ 0.05 (*p ≤ 0.05, **p ≤ 0.01) with respect to indicated mouse groups.

We next investigated the effect of BL on appendicular bones including femur and tibia. The 3D micro-architecture of proximal metaphysis of the femur and tibia showed that BV/TV and Tb.Th were decreased and Tb.Sp increased in ovx mice compared with sham, suggesting loss of trabecular bones, and BL treatment reversed all these ovx-induced changes (**Figures 6C–F**). Cortical bones of femur and tibia showed thinning due to ovx as periosteal area (T.Ar), periosteal perimeter (T.Pm), and cortical thickness (Cs.Th) were decreased compared with the sham, and BL treatment reversed these changes (**Figures 7A–D**). These data demonstrate that BL administration significantly improves the micro-architecture and histomorphometric parameters of both trabecular and cortical bones in osteoporotic mice.

### BL Enhances Both Bone Mineral Density and Mechanical Strength

**Figure 8:**
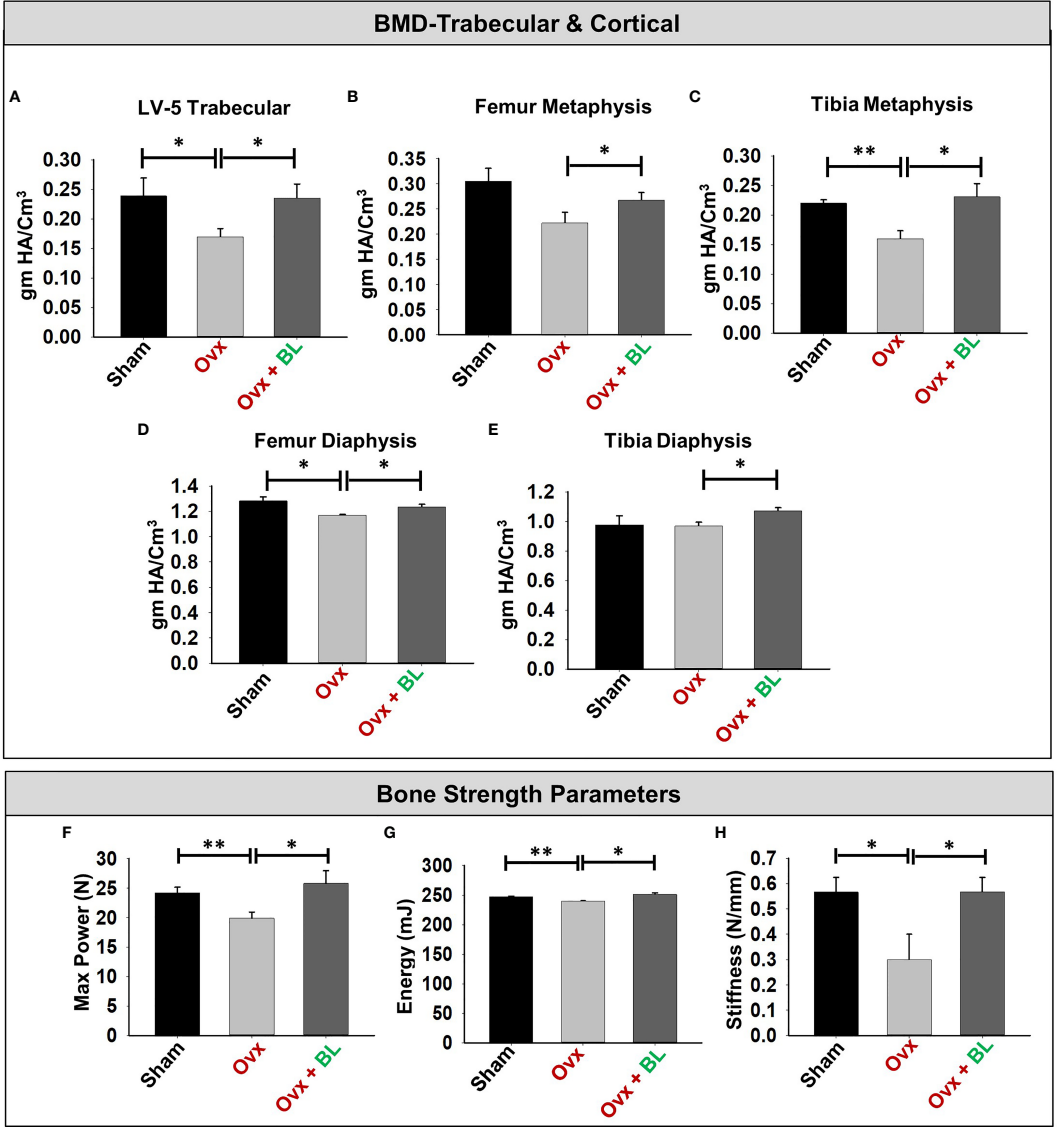
BL administration enhances bone mineral density (BMD) and mechanical strength of bones. **(A)** Graphical presentation of BMD of the LV-5 trabecular region, **(B)** femur metaphysis, **(C)** tibia metaphysis, **(D)** femur diaphysis, and **(E)** tibia diaphysis. **(F)** Three-point bending test of femur diaphysis representing max power (N), **(G)** energy (mJ), and **(H)** stiffness (N/mm). Data are reported as mean ± SEM. Similar results were obtained in two independent experiments with n = 6. Statistical significance of each parameter was assessed by ANOVA followed by paired group comparison. *p < 0.05, **p < 0.01 compared with indicated groups.

Since BMD is a predictor of osteoporotic fracture, we measured it at all weight-bearing bones of axial and appendicular sites. µCT allows the measurement of BMD, and our data showed that it was significantly decreased at all sites measured in ovx mice compared with sham; and BL treatment reversed these changes (**Figures 8A–E**).

We next assessed the bone quality by measuring the bending strength of femurs. The load-bearing capacity of bone, energy to failure, and stiffness were significantly decreased in the ovx mice compared with sham, and these parameters were maintained to the sham level in the BL group (**Figures 8F–H**). These data suggest maintenance of bone mass and strength in the ovx mice treated with BL.

### BL Promotes Bone Health by Modulating the Immunoporotic “Breg–Treg–Th17” Cell Axis

**Figure 9:**
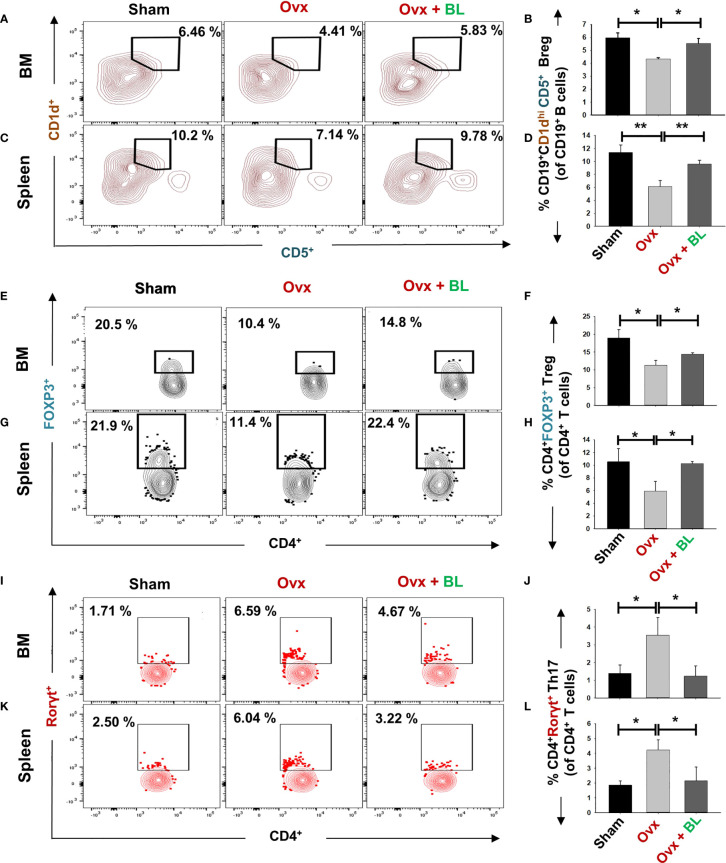
BL administration modulates Breg, Tregs, and Th17 cells *in vivo*. Cells from various lymphoid organs were harvested and analyzed for Bregs and Tregs. **(A)** Contour plots representing the percentages of CD19^+^CD1d^hi^ CD5^+^ Bregs in BM. **(B)** Bar graphs representing the percentages of CD19^+^CD1d^hi^ CD5^+^ Bregs in BM. **(C)** Contour plots representing the percentages of CD19^+^CD1d^hi^ CD5^+^ Bregs in spleen. **(D)** Bar graphs representing the percentages of CD19^+^CD1d^hi^ CD5^+^ Bregs in spleen. **(E)** Contour plots represent percentages of CD4^+^Foxp3^+^ Tregs in BM. **(F)** Bar graphs representing percentages of CD4^+^Foxp3^+^ Tregs in BM. **(G)** Contour plots represent percentages of CD4^+^Foxp3^+^ Tregs in spleen. **(H)** Bar graphs represent percentages of CD4^+^Foxp3^+^ Tregs in spleen. **(I)** Contour plots represent percentages of CD4^+^Rorγt^+^ Th17 cells in BM. **(J)** Bar graphs representing percentages of CD4^+^Rorγt^+^ Th17 cells in BM. **(K)** Contour plots are representing percentages of CD4^+^Rorγt^+^ Th17 cells in spleen. **(L)** Bar graphs representing percentages of CD4^+^Rorγt^+^ Th17 cells in spleen. Data are reported as mean ± SEM. Similar results were obtained in two independent experiments with n = 6. Statistical significance of each parameter was assessed by ANOVA followed by paired group comparison. *p < 0.05, **p < 0.01 compared with indicated groups.

**Figure 10:**
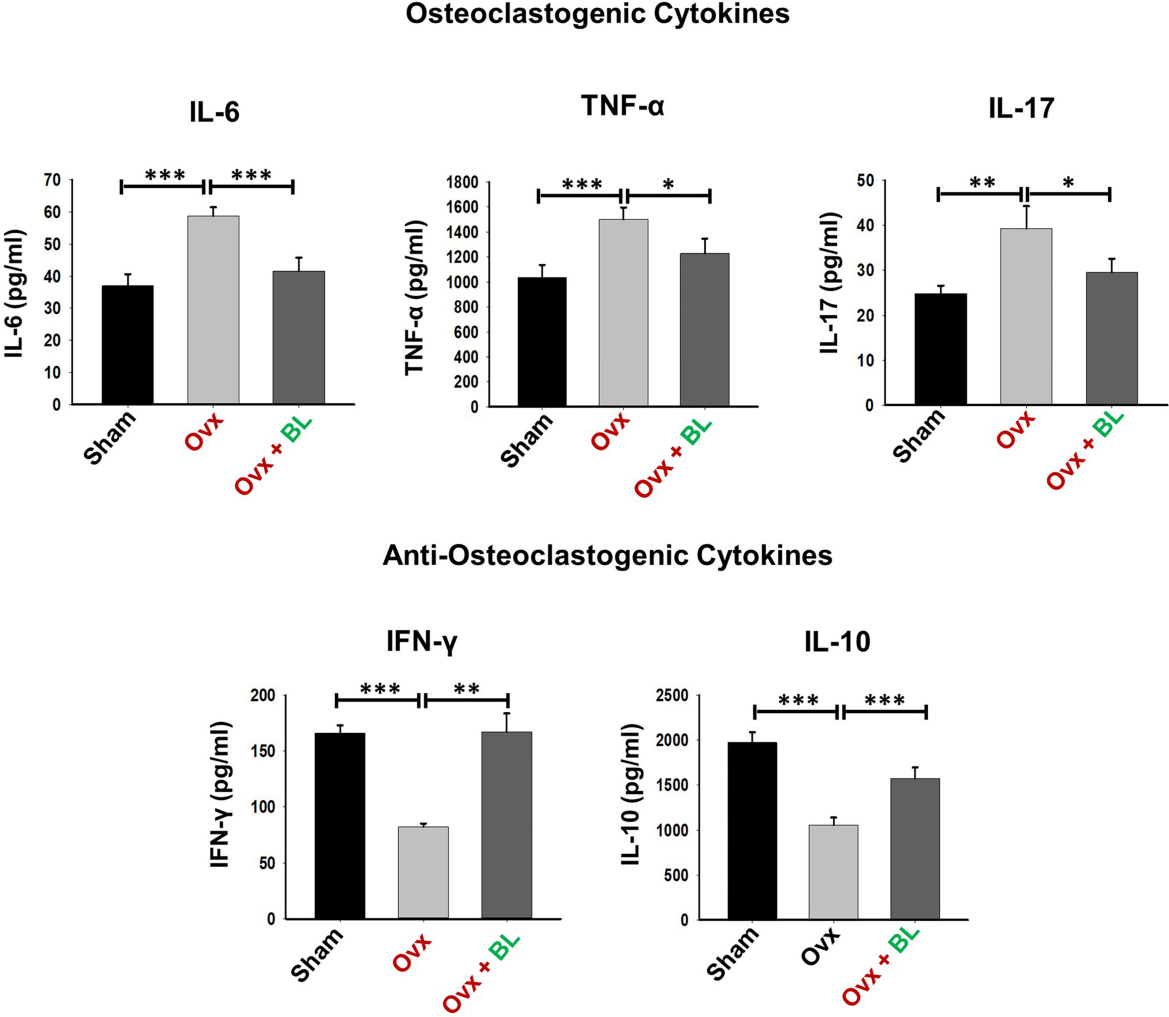
BL modulates cytokine balance in Ovx mice. Osteoclastogenic cytokines were analyzed in serum samples of mice by ELISA. Anti-osteoclastogenic cytokines were analyzed in serum samples of mice by ELISA. The results were evaluated by using ANOVA with subsequent comparisons by Student’s t-test for paired or non-paired data, as appropriate. Values are expressed as mean ± SEM (n = 6), and similar results were obtained in two independent experiments. Statistical significance was defined as p ≤ 0.05, *p ≤ 0.05, **p < 0.01, ***p ≤ 0.001 with respect to the indicated mouse group.

Our *in vitro* data showed that BL has anti-osteoclastogenic potential which is further augmented by the favorable modulation of the immunoporotic ability of Bregs. Thus, we next measured Breg, Treg, and Th17-cell populations in lymphoid organs including bone marrow (BM) and spleen in response to BL treatment in ovx mice. We observed that the CD19^+^CD1d^hi^CD5^+^ Breg population was significantly decreased in the BM (p < 0.05) and spleen (p < 0.01) of ovx mice compared with sham, and BL administration to ovx increased this cell population to the sham level (**Figures 9A–D**). Moreover, the percentages of CD4^+^Foxp3^+^ Tregs in BM (1.5-fold, p < 0.05) and spleen (2-fold, p < 0.05) in the ovx group were lower than those of the sham and BL treatment restored these parameters (**Figures 9E–H**). Conversely, ovx mice had higher percentages of CD4^+^Rorγt^+^ Th17 cells in BM (3-fold, p < 0.05) and spleen (3.5-fold, p < 0.05) than the sham, and BL treatment reversed these changes (**Figures 9I–L**). Besides, the circulating levels of pro-osteoclastogenic cytokines including IL-6, TNF-α, and IL-17 were increased and anti-osteoclastogenic cytokines such as IFN-γ and IL-10 were decreased in ovx mice compared with sham (**Figure 10**). BL treatment of ovx mice completely reversed the cytokine profiles, which further suggests its role in attenuating inflammatory bone loss in postmenopausal osteoporosis *via* modulating the immunoporotic “Breg–Treg–Th17” cell axis.

### BL Suppresses Osteoclastogenesis in Human PBMCs

**Figure 11:**
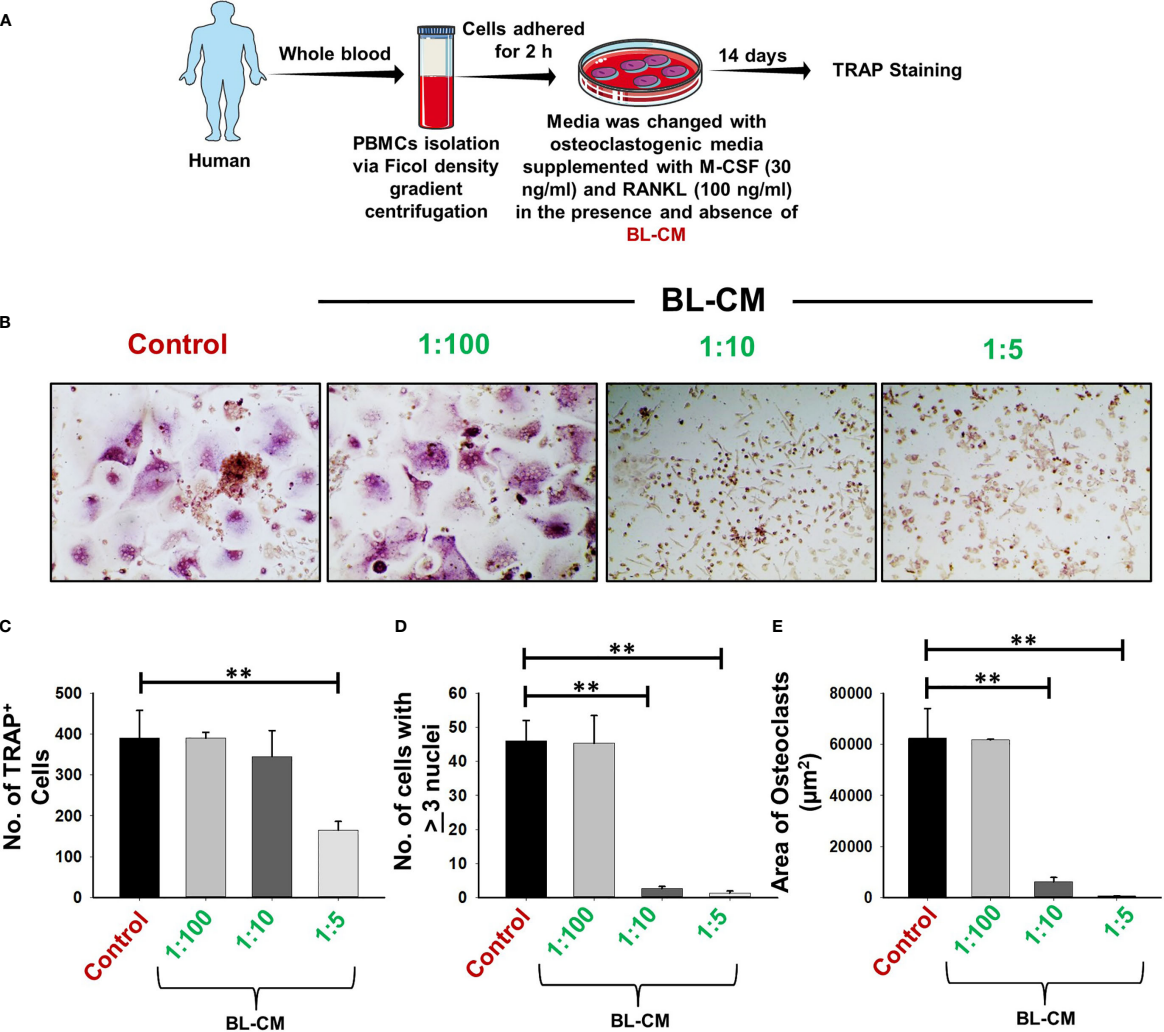
BL-CM suppress osteoclastogenesis in human PBMCs: **(A)** Osteoclast differentiation was induced in human PBMCs with M-CSF (30 ng/ml) and RANKL (50 ng/ml) with or without *Bifidobacterium longum*-conditioned media (BL-CM) at different ratios of 1:100, 1:10, and 1:5 for 14 days. Giant multinucleated cells were stained with TRAP, and cells with ≥3 nuclei were considered as mature osteoclasts. **(B)** Photomicrographs at ×20 magnifications were taken. **(C)** Number of TRAP-positive cells. **(D)** Number of TRAP-positive cells with more than 3 nuclei. **(E)** Area of osteoclasts. The above images are indicative of one independent experiment, and similar results were obtained in at least three different independent experiments. Statistical significance was considered as p  ≤  0.05 (**p  ≤  0.01) with respect to indicated groups.

Moving ahead, we corroborated our findings of the effect of BL in suppressing murine osteoclastogenesis in human samples. Human PBMCs when treated with BL-CM resulted in a concentration-dependent decrease in the number of multinucleated TRAP-positive cells (**Figures 11A–D**). Furthermore, area measurement using ImageJ software indicated significant reduction in the area of multinucleated osteoclasts in response to BL-CM (**Figure 11E**). These data thus confirm the anti-osteoclastogenic effect of BL on human PBMCs.

## Discussion

Osteoporosis is a chronic inflammatory condition resulting in an enhanced risk of developing fragility-related fractures at the site of the wrist, hip, and spine and is the 4th most burdensome chronic disease after ischemic heart disease, dementia, and lung cancer. This skeletal disorder affects predominantly women in comparison to men, and the risk increases with age. Currently, various pharmacological therapies are clinically used for the treatment of osteoporosis including bisphosphonates, teriparatide, denosumab, and romosozumab; however, various health concerns have been raised with their long-term administration. A study reported that the most prescribed osteoporosis drugs such as bisphosphonates and alendronate are found to be associated with the enhanced rate of developing depression and anxiety along with other adverse side effects (19). Thus, there is an exigent need to identify and develop safer therapies with minimal or no side effects. Experimental evidence suggests that nutritional supplementation including probiotics can maintain bone health in osteoporotic conditions. Thus, in the present study we investigated the immunoporotic potential of BL.

In cultures, *Lactobacillus rhamnosus* (LR) and *Lactobacillus reuteri* suppress the RANKL-mediated differentiation of osteoclast precursors into mature osteoclasts (16, 20). We observed that BL-CM also suppressed RANKL-induced osteoclastogenesis in murine and human precursor cells without affecting cell viability (**Figure S2**). Upon adhesion to the bone surface, polarization and reorganization of the cytoskeleton structure in osteoclasts leads to the generation of the F-actin ring structure. The organization of this dynamic F-actin structure is essential for resorptive functions of osteoclasts (21). Our imaging data from cultured cells show that the suppression of osteoclast formation accompanied the diminished ability of osteoclasts to remain functional. These findings were corroborated *in vivo* as resorptive lacunae and pits measured by SEM and AFM showed a significant increase in ovx mice compared with sham and BL treatment decreased it. Also, a reduction in the frequencies of osteoclast precursors in the BM (prime site of osteoclastogenesis) is further indicative of reduced osteoclast differentiation under *in vivo* conditions (**Figure S3**).

Under osteoporotic conditions, BL treatment resulted in bone conservation at both axial and appendicular sites and at both trabecular and cortical envelopes. Increased bone mass and cortical thickness in BL treatment appear to have contributed to increased resistance to bending failure of the femur over the ovx group. An improved trabecular microarchitecture in ovx mice treated with BL over ovx is likely to confer greater resistance to compressive strength, which however has not been measured.

Our data suggest that BL acts both *via* direct suppression of osteoclasts and by immunomodulation which results in an unfavorable environment for osteoclast formation and function. Additionally, our group along with others reported that probiotics such as *Lactobacillus rhamnosus*, *Lactobacillus casei*, *Lactobacillus acidophilus*, and *Bacillus clausii* enhance bone mass by regulating the intricate balance of “Treg–Th17” immune cells (10, 16, 17). However, till date, no study has ever reported the immunomodulatory potential of BL *via* its role on Tregs, Th17, and Bregs in regulating bone health under osteoporotic conditions. Building upon this evidence, we too were interested in evaluating the immunomodulatory potential of BL. Our flow cytometric data indicate that treatment with BL-CM significantly increased the differentiation of naïve T cells into Tregs along with simultaneous inhibition of Th17 cells under *in vitro* conditions (data not shown). In line with this, our *in vivo* data also confirm the immunomodulatory potential of BL in ovx mouse models. Accumulating evidence suggests that under both physiological and disease conditions, differentiation of Tregs and Th17 cells is regulated by the master regulator “Bregs” *via* IL-10, IL-35, and TGF-β cytokines (22, 23). Recently, we for the first time reported that Bregs possess strong osteo-protective potential in an IL-10-dependent manner, and any decrease in the frequencies of Bregs was directly linked with the pathophysiology of osteoporosis (7). Collectively, these studies suggest that modulation of Bregs would be a favorable therapeutic approach for the treatment of osteoporosis. Interestingly, our data from the present study suggest that BL treatment significantly enhanced the differentiation of splenic B cells into IL-10^+^ Bregs. Moreover, these BL-stimulated Bregs significantly enhanced the differentiation of Tregs along with the concurrently decreasing differentiation of Th17 cells when cocultured with BL-Bregs even under non-Treg–Th17-polarizing conditions. In addition, these BL-Bregs display enhanced anti-osteoclastogenic potential in comparison to non-BL-Bregs under *in vitro* conditions. Collectively, these critical experiments robustly establish the immunoporotic potential of BL-induced Bregs.

**Figure 12:**
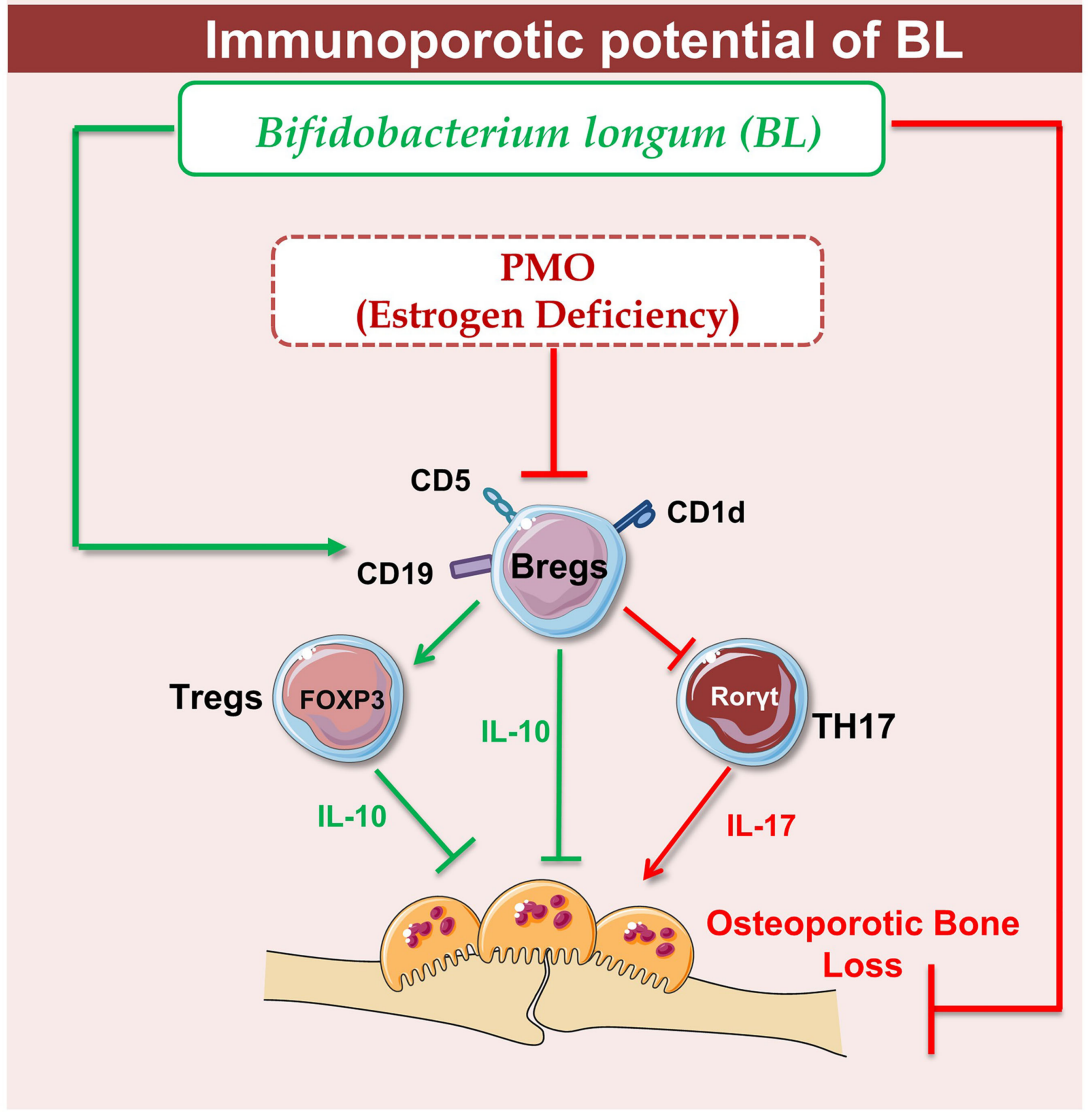
Summary of our results: Under physiological conditions, Bregs and Tregs inhibit osteoclastogenesis *via* producing the IL-10 cytokine whereas in osteoporotic conditions, dysregulation of the “Breg–Treg–Th17” cell axis promotes bone loss in osteoporotic conditions. Upon BL administration, enhancement of the Bregs population leads to maintenance of homeostatic balance between Tregs and Th17 cells that prevents the bone loss in osteoporotic conditions (image illustrated using Medical Art https://smart.servier.com/).

Supplementation of BL in ovx mice significantly enhanced bone health *via* modulating the nexus between Bregs, Tregs, and Th17 immune cells. Of note, it has been observed that the deficiency in estrogen hormones significantly enhanced the level of inflammatory cytokines such as IL-17 while substantially reducing the level of anti-osteoclastogenic cytokines such as IL-10, thereby augmenting bone loss in ovx mice. Importantly, our serum cytokine data further attest to the immunomodulatory potential of BL where administration of BL significantly reduced the levels of IL-17 cytokine (signature cytokine of Th17 cells) along with simultaneous induction of IL-10 cytokine (signature cytokine of Treg and Breg cells). Lastly, we decided to explore the possibility of our novel findings from preclinical models to clinical samples and observed that BL-suppressed RANKL induced osteoclastogenesis in human PBMCs. Collectively, our present study for the first time highlights the immunoporotic role of BL in skeletal homeostasis and emphasizes the probiotic BL as a novel osteoprotective agent for the treatment and management of osteoporosis. BL supplementation significantly enhances the BMD, bone strength, and micro-architecture of bones *via* modulating both the osteoclastogenesis and differentiation of Tregs, Bregs, and Th17 cells, thereby suggesting toward the pivotal role of the “Breg–Treg–Th17” cell axis in postmenopausal osteoporotic mouse models (**Figure 12**). However, the secretory metabolites responsible for the immunoporotic potential of BL are still warranted, thereby opening new avenues for further research.

## Data Availability Statement

The original contributions presented in the study are included in the article/**Supplementary Material**. Further inquiries can be directed to the corresponding author.

## Ethics Statement

The animal study was reviewed and approved by the Institutional Animal Ethics Committee of AIIMS, New Delhi, India (196/IAEC-1/2019).

## Author Contributions

RS contributed in the conceptualization and investigation of the study. LS, HD, NS, CS, and AB contributed to the methodology and formal analysis of data. KP helped in the µ-CT analysis. MM performed osteoclast culture in human PBMCs. LS developed the ovx mouse model. PM carried out the cytokine analysis. NC contributed in the µ-CT and bone mechanical strength data analysis. RS and LS contributed in the writing and editing of the manuscript. BV provided the valuable inputs. All authors reviewed the manuscript. All authors contributed to the article and approved the submitted version.

## Funding

This work was financially supported by the following projects: DST-SERB (EMR/2016/007158) and DBT (BT/PR41958/MED/97/524/2021), Govt. of India, and the intramural project from All India Institute of Medical Sciences (AIIMS, AI-798), New Delhi-India, sanctioned to RS. NC acknowledges funding from the Council of Scientific and Industrial Research, Govt. of India (MLP-2035). LS, HD, NS, CS, AB, BV, and RS acknowledge the Department of Biotechnology AIIMS, New Delhi-India, for providing infrastructural facilities. LS thanks UGC, NS thanks DBT, and AB thanks DST SERB for the research fellowship.

## Conflict of Interest

The authors declare that the research was conducted in the absence of any commercial or financial relationships that could be construed as a potential conflict of interest.

## Publisher’s Note

All claims expressed in this article are solely those of the authors and do not necessarily represent those of their affiliated organizations, or those of the publisher, the editors and the reviewers. Any product that may be evaluated in this article, or claim that may be made by its manufacturer, is not guaranteed or endorsed by the publisher.

## Supplementary Material

Supplementary Figure 1 Body weight of Mice. Mice were divided into 3 groups viz. Sham, Ovx and Ovx + BL group that received BL at 10^9^ CFU/day orally reconstituted in drinking water. At the end of 45 days, mice were sacrificed and analysed for various parameters and body weight was monitored at regular intervals; Values are reported as mean ± SEM (n = 6).

Supplementary Figure 2 Cell cytotoxicity assay. Bone marrow cells (BMCs) were treated with different dilutions of BL supernatant for 48 h and MTT assay was performed for evaluating the cell cytotoxic effects of BL.

Supplementary Figure 3 BL administration reduces F4/80^+^ osteoclasts precursors in BM. **(A)** Histograms representing percentages of macrophages in all the groups **(B)** Graphical representation of percentages of macrophages in three groups. The results were evaluated by using ANOVA with subsequent comparisons by Student t-test for paired or non-paired data, as appropriate. Values are expressed as mean ± SEM (n = 6) and similar results were obtained in two independent experiments. Statistical significance was defined as p ≤ 0.05, *p ≤ 0.05, **p < 0.01 ***p ≤ 0.001 with respect to indicated mice group.

## Abbreviations

Bregs, regulatory B cells; RANKL, receptor activator of nuclear factor kappa B ligand; LPS, lipopolysaccharide; IL, interleukin; TGF, transforming growth factor; RA, rheumatoid arthritis; SLE, systemic lupus erythematosus; PMA, phorbol 12-myristate 13-acetate; BMCs, bone marrow cells; M-CSF, monocyte colony-stimulating factor; TRAP, tartrate-resistant acid phosphatase; BMD, bone mineral density; SEM, scanning electron microscopy; AFM, atomic force microscopy; Tregs, regulatory T cells.
