# Supplementary figures and images for: Bifidobacterium longum Ameliorates Ovariectomy-Induced Bone Loss via Enhancing Anti-Osteoclastogenic and Immunomodulatory Potential of Regulatory B Cells (Bregs)
Source: Front Immunol. 2022 May 25;13:875788. doi: 10.3389/fimmu.2022.875788 (PMC9174515; doi:10.3389/fimmu.2022.875788)

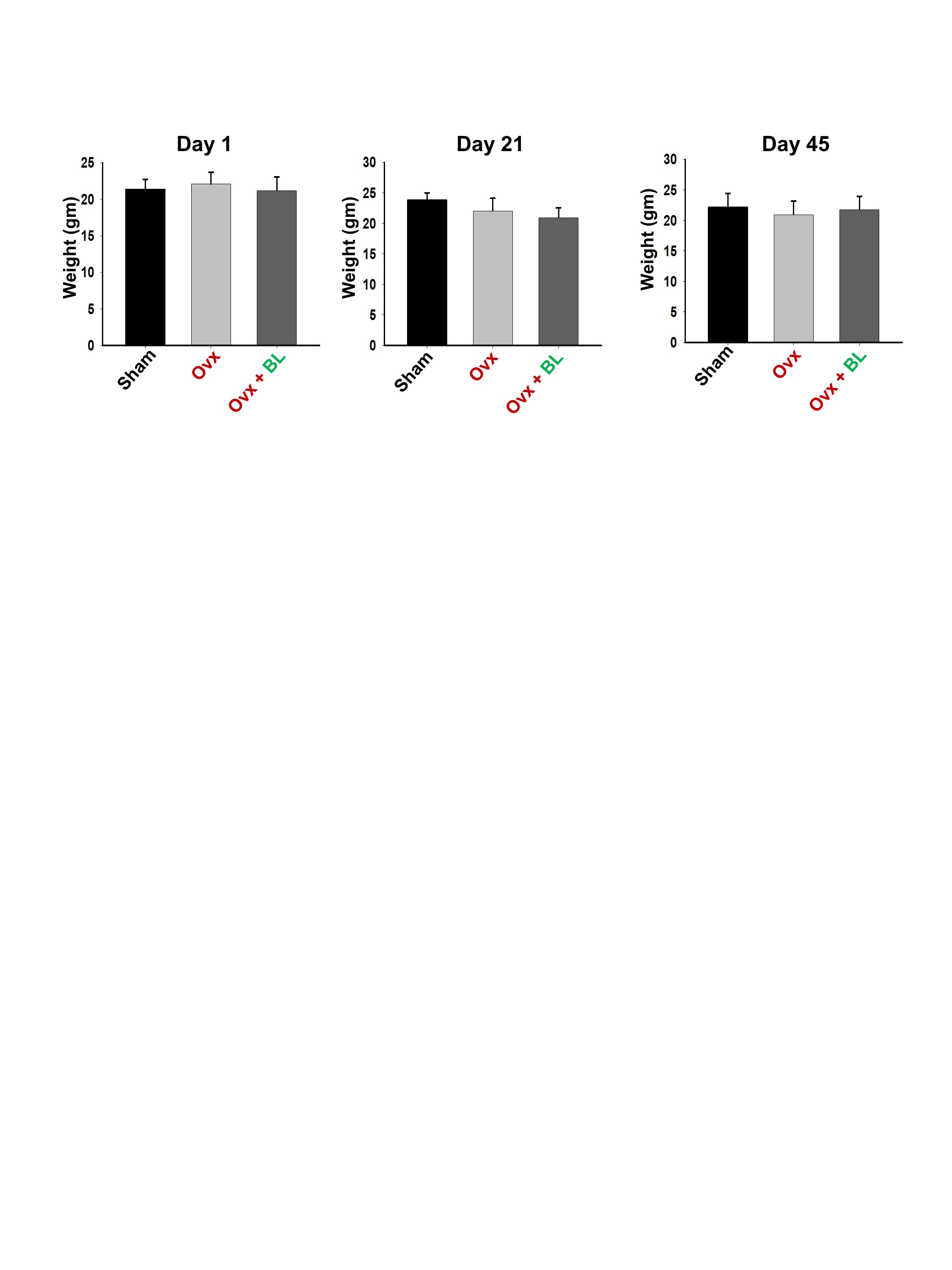

Supplement: Supplementary Figure 1 — Body weight of Mice. Mice were divided into 3 groups viz. Sham, Ovx and Ovx + BL group that received BL at 109 CFU/day orally reconstituted in drinking water. At the end of 45 days, mice were sacrificed and analysed for various parameters and body weight was monitored at regular intervals; Values are reported as mean ± SEM (n = 6). [file Image_1.jpg]

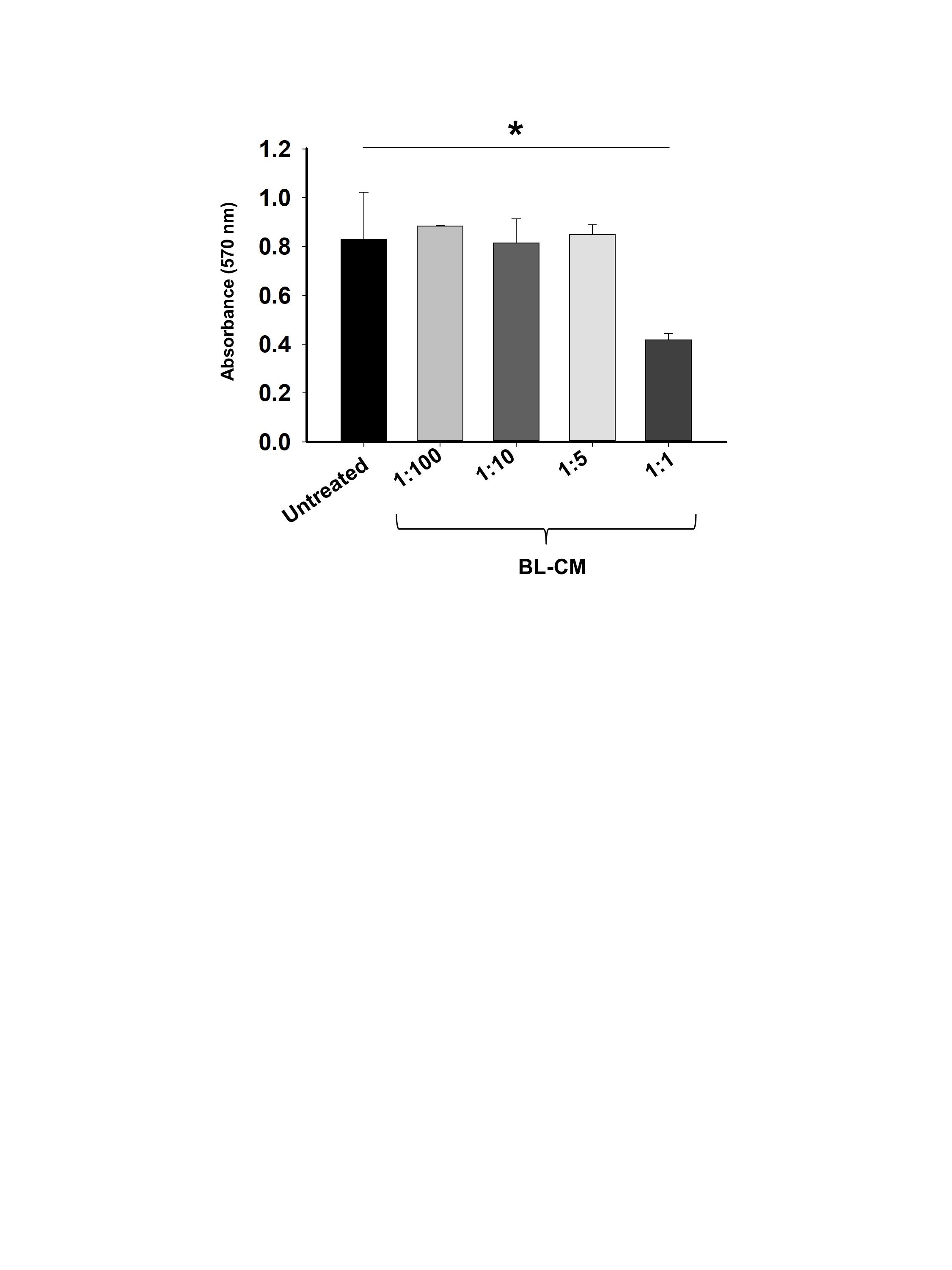

Supplement: Supplementary Figure 2 — Cell cytotoxicity assay. Bone marrow cells (BMCs) were treated with different dilutions of BL supernatant for 48 h and MTT assay was performed for evaluating the cell cytotoxic effects of BL. [file Image_2.jpg]

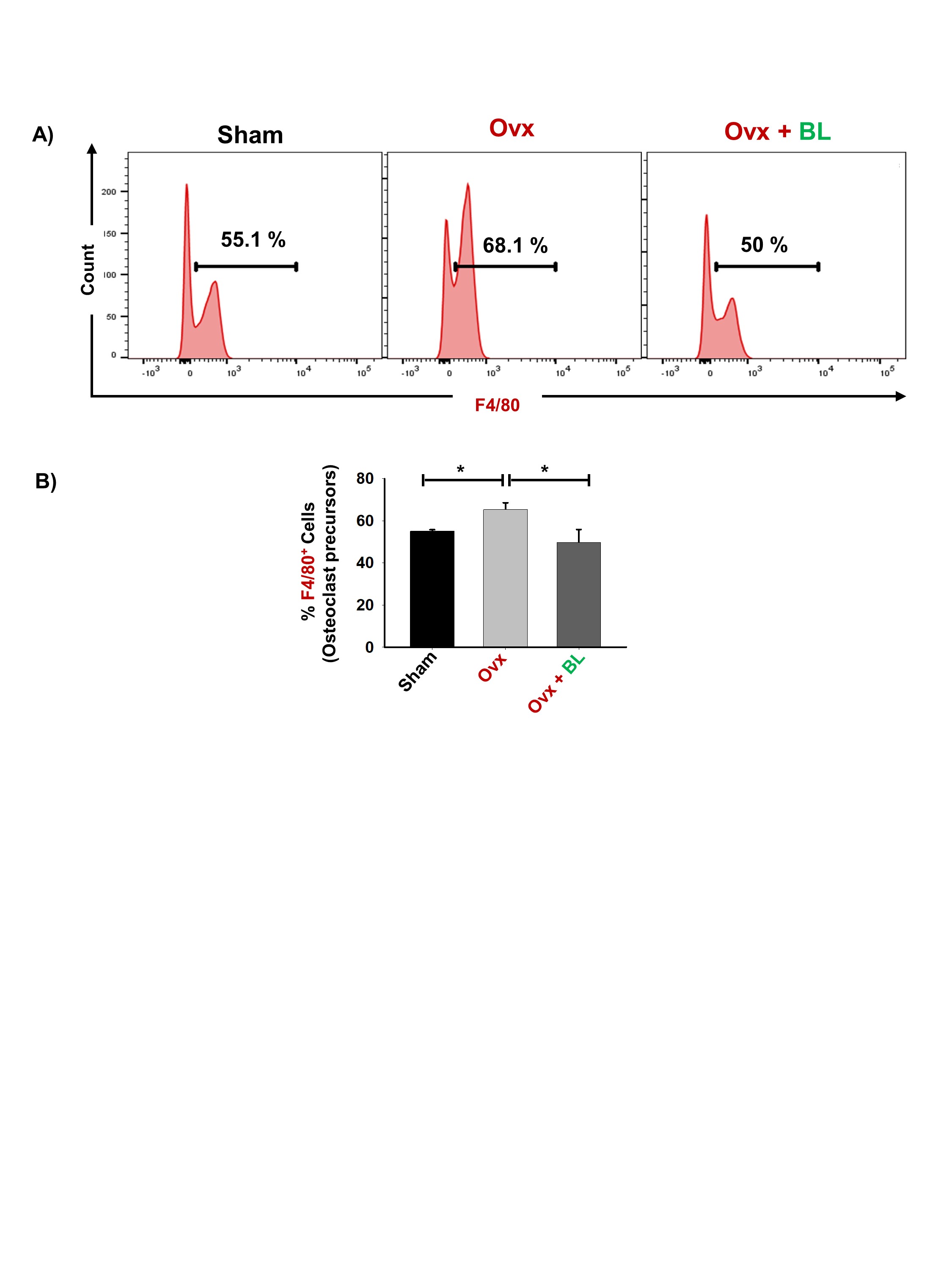

Supplement: Supplementary Figure 3 — BL administration reduces F4/80+ osteoclasts precursors in BM. (A) Histograms representing percentages of macrophages in all the groups (B) Graphical representation of percentages of macrophages in three groups. The results were evaluated by using ANOVA with subsequent comparisons by Student t-test for paired or non-paired data, as appropriate. Values are expressed as mean ± SEM (n = 6) and similar results were obtained in two independent experiments. Statistical significance was defined as p ≤ 0.05, *p ≤ 0.05, **p < 0.01 ***p ≤ 0.001 with respect to indicated mice group. [file Image_3.jpg]
